# Pomegranate juice fermented by tannin acyl hydrolase and *Lactobacillus vespulae* DCY75 enhance estrogen receptor expression and anti-inflammatory effect

**DOI:** 10.3389/fphar.2022.1010103

**Published:** 2022-09-29

**Authors:** Reshmi Akter, Jong Chan Ahn, Jinnatun Nahar, Muhammad Awais, Zelika Mega Ramadhania, Se-Woung Oh, Ji-Hyung Oh, Byoung Man Kong, Esrat Jahan Rupa, Dong Wong Lee, Deok Chun Yang, Se Chan kang

**Affiliations:** ^1^ Graduate School of Biotechnology, College of Life Sciences, Kyung Hee University, Seoul, Gyeonggi-do, South Korea; ^2^ SMART FRUIT CO., LTD., Guri, Gyeonggi-do, South Korea; ^3^ Fruitycompany Co., Ltd., Guri, Gyeonggi-do, South Korea; ^4^ Department of Oriental Medicinal Biotechnology, College of Life Sciences, Kyung Hee University, Seoul, Gyeonggi-do, South Korea; ^5^ Hanbangbio Inc., Suwon, Gyeonggi-do, South Korea

**Keywords:** *Lactobacillus vespulae*, fermentation, menopause, pomegranate, inflammation

## Abstract

Phenolics are phytochemicals in plants, fruits, and vegetables have potential health-promoting efficacies. However, mostly available as a complex form. So, to increase the contents and nutritional value of the phenolic compounds, fermentation is most readily used in the food industry. Especially, the hydrolyzable tannins present in the pomegranate that can be liberated into monomolecular substances, which enhances biological activity. Thus, this study aims to convert hydrolyzable tannins to ellagic acid by fermentation using Tannin acyl hydrolase (TAH) and a novel bacteria strain *Lactobacillus vespulae* DCY75, respectively to investigate its effect on Estrogen receptor alpha (ERα) and estrogen receptor beta (ERβ) mRNA expression along with inflammation inhibition. As a result, the fermentation enhanced the ellagic acid content up to 70% by the synergetic effect of TAH and DCY75. Furthermore, fermented pomegranate (PG-F) increased cellular proliferation as well as upregulated the gene expression of estrogen regulators such as ERα, ERβ, and pS2 in breast cancer cell line (MCF-7), which commonly used to evaluate estrogenic activity. Moreover, to study the inflammation associated with low estrogen in menopause, we have analyzed the inhibition of nitric oxide (NO)/inducible nitric oxide synthase (iNOS) in RAW 264.7 cells. The PG-F juice did not exert any cytotoxicity in RAW 264.7 cells and inhibited NO production along with the downregulation of a major pro-inflammatory cytokine iNOS which indicates the anti-inflammatory potential of it. To sum it up, the fermented commercial pomegranate juice using a novel bacteria strain increased the amount of ellagic acid that the value added bioactive of pomegranate and it has significantly increased the estrogenic activity via upregulating estrogen related biomarkers expression and reduced the risk of related inflammation via NO/iNOS inhibition. This study could be a preliminary study to use fermented pomegranate as a potential health functional food after further evaluation.

## Graphical Abstract

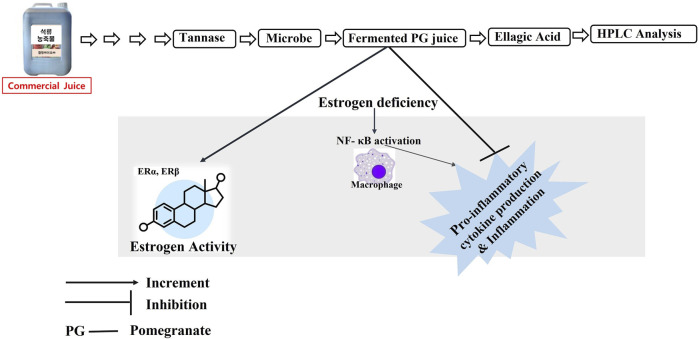

## 1 Introduction

Estrogens as essential steroid hormones, are secreted mainly from the ovary and placenta and have indispensable roles in women’s reproductive development. It affects the proliferation, differentiation, and physiological tasks of reproductive organs, including the urinary tract, oviduct, mammary gland, and vagina (Mc Rodrigues et al., 2018). Estrogen is also responsible for performing a significant role in non-reproductive organs such as the immune, skeletal, cardiovascular, and nervous systems (Ikeda et al., 2019). Moreover, low estrogen level may lead to an irregular menstrual cycle associated with menopause or premature menopause (Talaulikar, 2022). Consequently, less ovarian estrogen production during menopause causes hot flushes, night sweats, vaginal dryness, insomnia, changes in metabolism, sexual dysfunction, and physical complications such as depression, mood swings, skin changes, etc. (Secoșan et al., 2019; Hirsch and Manson 2022). Plenty of reports claim that inflammation can increase during menopause due to declining estrogen level (Au et al., 2016; Skoczek-Rubińska et al., 2021). To minimize menopausal complications, the use of synthetic hormones (estrogen or progesterone) that imitate the function of endogenous hormones has become a priority since the 1970s (Mc Rodrigues et al., 2018). Estrogen interacts with two types of estrogen receptors (ERα and ERβ) to exert its beneficial biological effects (Kim et al., 2016). Much evidence has supported the success of estrogen replacement therapy (ERT); however, long-term use of ERT has specific side effects, including weight gain, depression, cancer development, vaginal bleeding, and stroke (Anagnostis et al., 2016; Angioli et al., 2018). Therefore, complementary remedies using herb-based phytoestrogen have become a hotspot research topic in this day and age. Phytoestrogens are plant-derived estrogens that include isoflavones, phenolic compounds, lignans, coumestans, and flavonoids. Phytoestrogen has a similar structure to human estrogen thus it can bind with ERs receptors (Chang et al., 2018). The use of synthetic estrogen may increase the risk of endometrial cancer, myocardial infarction, vaginal bleeding and invasive breast cancer in post-menopausal women (Liang and Shang 2013; Delgado and Lopez-Ojeda 2021), but such risks have not been proven while consuming plant-based phytoestrogens (Glazier and Bowman 2001; Tempfer, Froese et al., 2009). Moreover, much research has found the beneficial effect of phytoestrogen against cancer (Barnes and Peterson. 1995), cardiovascular diseases (Sirotkin and Harrath 2014), obesity, and skin diseases, including immune system (Desmawati and Sulastri 2019) though further studies are needed to specify consumption quantity or type of phytoestrogens.

*Punica granatum* Linn. Belongs to Punicaceae family and follows the name *Malum granatum* commonly identified as pomegranate, which is a native fruit in the Middle East. This fruit is intensively used in the folk medicine of innumerable traditions (Li et al., 2006). *P. granatum* (PG) is distributed throughout Iran and the Himalayas in northern India, Malaysia, tropical Africa, Japan, China, Russia, and the drier parts of Southeast Asia, including some parts of the United States (Fadavi et al., 2006). PG is consumed as fresh fruit. The edible parts of pomegranate fruits are used to prepare fresh juice, canned beverages, jelly, jam, and for flavoring and coloring beverage products (Viuda-Martos et al., 2010). Different parts of this fruit are applicable for food, medicine, pharmaceuticals, cosmetics, and nanotechnology; therefore, it is considered a superfood (Putnik et al., 2019; Puneeth and Chandra 2020). According to the research conducted by Wang et al. (2018), PG is rich in hydrolyzable tannins precursor of ellagic and gallic acid, which play a crucial role in the physiological activity of Pomegranate. In addition, a previous report claimed that ellagic acid is a natural selective estrogen receptor modulator (Papoutsi et al., 2005). Bioconversion of tannin has become a hotspot of scientific research due to its commercial significance, strengthened into a glassy state with fast absorption, and scientific relevance to end products (Zhang et al., 2009). Fungal and bacterial organisms can transform hydrolyzable tannin through Tannase (Tannin acyl hydrolase), a key enzyme capable of hydrolyzing ester and depside bonds. In the contemporary study, we used a novel bacteria strain named *Lactobacillus vespulae* DCY75 introduced by HanbangBio laboratory, Kyung Hee University, for fermentation along with Tannin acyl hydrolase (TAH). *Lactobacillus* is a highly useful microbe as well as readily available. *Lactobacillus vespulae* DCY75 is a novel strain isolated from the gut of a queen wasp (Vespula vulgaris) by HanbangBio lab (Hoang et al., 2015), Kyung Hee University, and was used to convert PG precursors into ellagic acid. Both enzymes and bacteria are used together for the fermentation of PG juice to increase the ellagic acid content by synergistic effect.

Pomegranate has been used in medicinal systems to combat diseases such as diarrhea, ulcers, diabetes, and cancer (Saxena and Vikram, 2004, 30 Kumari et al., 2012, 31 Khwairakpam et al., 2018). Due to polyphenols high content, hydrolyzable tannins, anthocyanins, and Pomegranate have shown more excellent anti-inflammatory activity and antioxidant activity than Vitamin E, *β*-carotene, and ascorbic acid (Sharma et al., 2017). As fruit juices are known as a regular functional drink in the market sector, it is essential to make sure the full benefits of the juice are attributed to probiotics are to be experienced. In a previous report, the production of probiotic pomegranate juice through its fermentation by strains of lactic acid bacteria: *Lactobacillus plantarum*, *L. delbruekii*, *L. paracasei*, *L. acidophilus* was examined (Mousavi et al., 2011). However, the fermentation of PG juice using TAH enzyme and novel strain *Lactobacillus vespulae* DCY75 is not reported yet. Therefore, this study evaluated the increment of polyphenol content by fermentation along with estrogen-like effects and anti-inflammatory activity of fermented Pomegranate (PG-F) juice *in vitro*, along with the potential mechanisms.

## 2 Materials and methods

### 2.1 Plant material

The Clear pomegranate concentrate (65 brix, pH 4.4–5.4) was obtained from Fruit Tech Natural S. A. (Murcia, Spain).

### 2.2 Optimum condition of tannase treatment

Tannase (Tannin acyl hydrolase, TAH) was obtained from Kikkoman Biochemifa (Nishi-Shinbashi, Japan). A 250 ml conical flask was used for tannase treatment. To prepare 5 brix pomegranate juice, the clear pomegranate juice was diluted with distilled water. The optimization of the enzyme was carried out at first. Tannase (500 unit/g) was mixed with 100 ml of 5brix pomegranate juice (pH = 5.4) to a concentration of 0.01, 0.05, 0.1, 0.25, 0.5% respectively. Then it was incubated at 37°C with shaking (150rpm) for 2 h. Secondly, the optimization of incubation time was carried out. Tannase (500 unit/g) was mixed with 100 ml of 5brix pomegranate juice to a concentration of 0.1%. And incubation was carried out at 37°C for 0.5, 1, 2, 3, 4 h. After incubation, dependent on enzyme concentration and time, tannase in the mixture was inactivated by storage in the deep freezer (−80°C). The aliquot was filtered with 0.45 μm syringe filter into a 2 ml screw top vial before loading on HPLC system.

### 2.3 Inoculum preparation and culture condition of *Lactobacillus vespulae* DCY 75

The *Lactobacillus vespulae* DCY75T (KCTC 21023T) used in this study were obtained from Ginseng Bank (Suwon, Korea). For seed culture, the *L. vespulae* DCY75 was grown on MRS agar plates for 1 day. A single colony was selected from the plate and inoculated in MRS broth. The Incubation of cultured strain was carried out at 30°C for 1 day.

### 2.4 Fermentation of pomegranate juice

The fermentation of pomegranate juice was carried out using a 50 ml tube. The total reaction volume was 20 ml. The 0.2 ml of *L. vespulae* DCY75 (approximately 107–8 cfu/ml) was inoculated into 19.8 ml five Brix pomegranate juice (pH 5.4). Then the tannase (500unit/g) was added in the mixture to the concentration of 0.1%. The final mixtures were then incubated at 30°C with shaking (150rpm) for 2 days. After incubation, DCY 75 strain and tannase in the mix were inactivated. Then the mixture was centrifuged at 8,000 rpm for 15 min, and the supernatant was filtered with a 0.45 μm syringe prior to HPLC analysis. Instead of DCY 75 strain, 0.2 ml distilled water was mixed with 19.8 ml of 5brix pomegranate juice without tannase as control, followed by the fermentation mentioned above procedure.

### 2.5 High-performance liquid chromatography system and condition for analyzing chemical contents

For performing the high-performance liquid chromatography (HPLC), Sun et al. (2021) method was followed with minor modification. The PG juice was filtered through a 0.45 µm syringe after centrifugation. The conditions of the HPLC system for analyzing phenolic compounds are shown in (Table 1).

**TABLE 1:** The conditions of the HPLC system for analyzing phenolic acids.

System/Condition	Phenolic acids (gallic acid and ellagic acid)
Flow rate	1.0 ml/min
Wavelength	260 nm
Injection Volume	5 µl
Solvents	Gradient eluent: A: Methanol B: 0.1% acetic acid in water
Column Temperature	35°C

HPLC system consists of Agilent 1,260 Infinity Variable Wavelength Detector (G1314F), Agilent 1,260 Infinity Standard Autosampler (G1329B), Agilent 1,260 Infinity Column Thermostat Compartment (G1316A), and the Agilent 1,260 Infinity Quaternary Pump (G1311B). ZORBAX Eclipse Plus C18 column (250 mm × 4.6 mm, 5 µm particle size) (Milford, MA, United States) was chosen as a stationary phase. For phenolic acids analysis, the eluent compositions were as follows: (0–8 min, 90%–80% B; 8–30 min, 80%–55% B; 30–60 min, 55%–30% B).

### 2.6 Total phenolic and total flavonoid contents determination

Total phenolics and total flavonoids of each sample were determined using the Folin–Ciocalteu method with slight modifications (Huo et al., 2021). 0.5 g of dried powdered material was extracted using 20 ml 80% methanol for 1 h with three times repetitions, then the filtrate was combined together for evaporation. For further compound analysis, the crude extract was redissolved in distilled water. To analyze total phenolics, 0.3 ml of PG juice was added to 1.5 ml Folin-Ciocalteu reagent in corresponding wells of a 96-well microplate and incubated for 5 min after shaking thoroughly. Then 1 ml of 7.5% Na2CO3 solution was added to the microplate, and the mixture was kept in the dark for 30 min. Finally, the absorbance was quantified at 715 nm. Total phenolic content was evaluated from a standard curve using gallic acid as the standard. Results were expressed as µmol gallic acid equivalent per Gram of dry weight (µmol GAE/g DW).

Total flavonoid content was measured by using the reaction mixture containing 0.3 ml of PG juice, 0.3 ml 5% NaNO2, and 0.3 ml 10% AlCl3. The well-mixed mixture was allowed to incubate for 6 min, followed by the addition of 0.5 ml 1 N NaOH. After mixing the solution well, the absorbance was immediately measured at 510 nm. Total flavonoid content was calculated with a calibration curve based on rutin, and the results were expressed as µmol rutin equivalent per Gram of dry weight (µmol RE/g DW).

### 2.7 Assessment of radical scavenging activities

The free radical scavenging activity was evaluated using DPPH method with minor modifications to previous method (Akter et al., 2021). 20 µl of extract and 180 µl of DPPH solution were added to a 96 well plate and then incubated in the dark for 30 min at 25°C, followed by vigorous shaking. Afterward, the absorbance was measured at 517 nm. The percentage inhibition of the samples was assessed by using a formula mentioned below:

(1-Absorbance of sample/Absorbance of control) * 100.

The reducing power activity of the samples were determined using 100 µl of samples with 250 µL of phosphate buffer with a pH 6.6 and 250 µl of (1%) potassium ferricyanide. Then the mixture was incubated at 40°C in a water bath for 20 min. Then the mixture was cooled down and 250 µl of (10%) trichloroacetic acid was added. The mixture was centrifuged at 8,000 rpm for 10 min, and supernatant was added with 100 µL distilled water and 20 µl of instantly prepared (0.1%) ferric chloride solution. The absorbance was determined at 700 nm. A blank was performed without adding PG samples. Ascorbic acid and gallic acid were applied as standards, and the results were expressed in mg of ascorbic acid and gallic acid equivalents per Gram (mg AAE/g DW or mg GAE/g DW) of the sample.

### 2.8 Cell culture

MCF-7, an ER-positive human breast cancer cell line, and macrophage cell line RAW 264.7 were purchased from American Type Culture Collection (ATCC). MCF-7 cells were cultured in DMEM (containing 4,500 mg/L D-glucose, L-glutamine, sodium pyruvate, and sodium bicarbonate) medium supplemented with 10% (V/V) charcoal-stripped fetal bovine serum (FBS) and penicillin-streptomycin solution. RAW 264.7 cells were cultured in DMEM with 10% FBS, and 1% p/s. The cells were grown at 37°C in a humidified atmosphere of 95% air/5% CO2. DMEM was purchased from Welgene (Daegu, Korea), FBS and P/S were purchased from GenDEPOT, while Charcoal-Dextran was bought from Sigma-Aldrich Chemicals, United States 17β-Estradiol was purchased from Sigma (Louis, MO, United States).

### 2.9 Cell proliferation assay

The cell proliferation assay was performed according to the protocol reported by (Lim, Ha et al., 2011) with slight modifications. MCF-7 cells were cultured in a hormone-free medium, seeded at a density of 1 × 10^4^ cells/well in 96-well plates, and allowed to grow overnight at 37°C in a 5% CO2 incubator. After discarding the medium, cells were separately treated with17β-Estradiol and Pomegranate. Then the cells were incubated for 24 h, and cell viability was detected using MTT (3-[4,5-dimethylthiazol-2-yl]-2,5-diphenyltetrazolium bromide) solution (20 µl/well) for 2–3 h. Finally, the cells were stained using 100 µl DMSO to produce formazan crystal into a colored solution. The absorbance was measured at 570 nm using a microplate reader (BioTek Instruments, Inc. Winooski, VT, United States).

### 2.10 Determination of nitrite levels

RAW 264.7 cells were used to measure cellular nitric oxide levels. For this, cells were pretreated with different concentrations of pomegranate juice, followed by the stimulation with 1 μg/ml LPS. Then the cells were kept in an incubator for 24 h. Griess reagent was used to quantify the nitrite level in the medium. Concisely, 100 μl of supernatant was mixed with 100 μl of Griess reagent. Finally, the absorbance was measured using a microplate reader (Bio-Tek Instruments, Inc.) at 540 nm (Akter et al., 2022).

### 2.11 Gene expression analysis

MCF-7 cells were seeded in 12 well plate at a density of 0.5 × 10^6^ cells/well and cultured as mentioned in Section 2.8. The medium was then replaced with phenol red- and serum-free DMEM with or without PM and FPM (100 μg/ml). After incubation for 24 h, cells were washed twice with PBS. To analyze the reverse transcription polymerase reaction (RT-PCR), total RNA was extracted from pomegranate-treated MCF7 cells with TriZol LS reagents (Invitrogen, Carlsbad, CA, United States). After that, cDNA was synthesized following the recommended protocol of a commercial cDNA synthesis kit (Onebio, Lithuania, EU). For cDNA synthesis, 1 µg of total RNA was used. The mentioned conditions were applied for cDNA synthesis: 42°C for 1 h and then 72 °C for 5 min.

Then the synthesized cDNA was used for amplification of the targeted gene. The list of primers used for the RT-PCR is mentioned in Table 2.

**TABLE 2:** The list of primers used for the RT-PCR.

Genes	Forward primers	Reverse primers
ERα	CCG​CTC​ATG​ATC​AAA​CGC​TCT​AAG	GCC​CTC​TAC​ACA​TTT​TCC​CTG​GTT (Farabegoli, Barbi et al., 2007)
pS2	ATG​GCC​ACC​ATG​GAG​AAC​AA	ATA​GAA​GCA​CCA​GGG​GAC​CC (Farabegoli, Barbi et al., 2007)
ERβ iNOS	TTC​CCA​GCA​ATG​TCA​CTA​ACT T ACC​CAA​GGT​CTA​CGT​TCA​GG	TTG​AGG​TTC​CGC​ATA​CAG​A (Kim, Kim et al., 2016) CGC​ACA​TCT​CCG​CAA​ATG​TA (Ahn, Siddiqi et al., 2016)
GAPDH	AAT​GGG​CAG​CCG​TTA​GGA​AA	GCGCCCAATACGACCAAA (Castro-Aceituno, Ahn et al., 2016)

For the PCR amplifications following conditions were used: 94°C for 5 min for 1 cycle and then 94°C for 1 min, 56°C for 30 s and 72°C for 1 min for 30 cycles. Data analysis was performed with ImageJ1.30v software (Simova-Stoilova, Vaseva et al., 2010; Hazman, 2022). The relative gene expression levels were normalized to the expression of the housekeeping gene (GAPDH).

### 2.12 Statistical analysis

All data were expressed as mean ± SE of at least three independent experiments. All analyses were performed using GraphPad Prism (GraphPad software, La Jolla, CA, United States). The total variations between treated groups and untreated (control) groups were determined by Student’s t-test and two-way analysis of variance (ANOVA). The significant difference was accepted at a level of *p* < 0.05.

## 3 Result and discussion

### 3.1 Optimization tannase treatment

**FIGURE 1:**
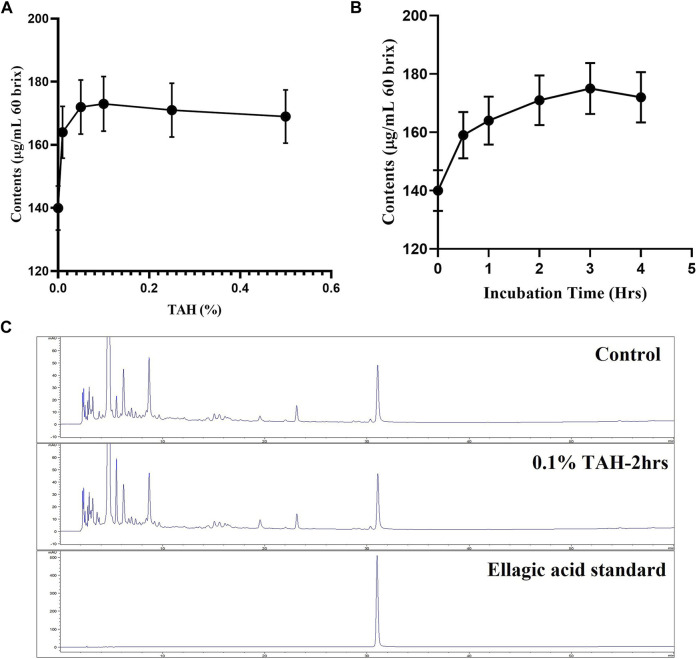
Optimization of Tannase treatment. **(A)** Condition of enzyme percentage **(B)** incubation time **(C)** HPLC analysis for Tannase treatment.

Tannin acyl hydrolase rich is an inducible enzyme produced by various microorganisms. In current study, TAH originated from *Aspergillus oryzae* and was applied to convert active substance present as a precursor (ellagitannins) into ellagic acid. Pomegranate juice was treated with TAH for the enhancement of ellagic acid. And the condition of enzyme percentage and time used for the fermentation was optimized. The result revealed that 0.1% enzyme showed the highest ellagic acid production, whereas 2 h was the optimal time for the best ellagic acid production (Figure 1).

### 3.2 Analysis of ellagic acid using High-performance liquid chromatography

**FIGURE 2:**
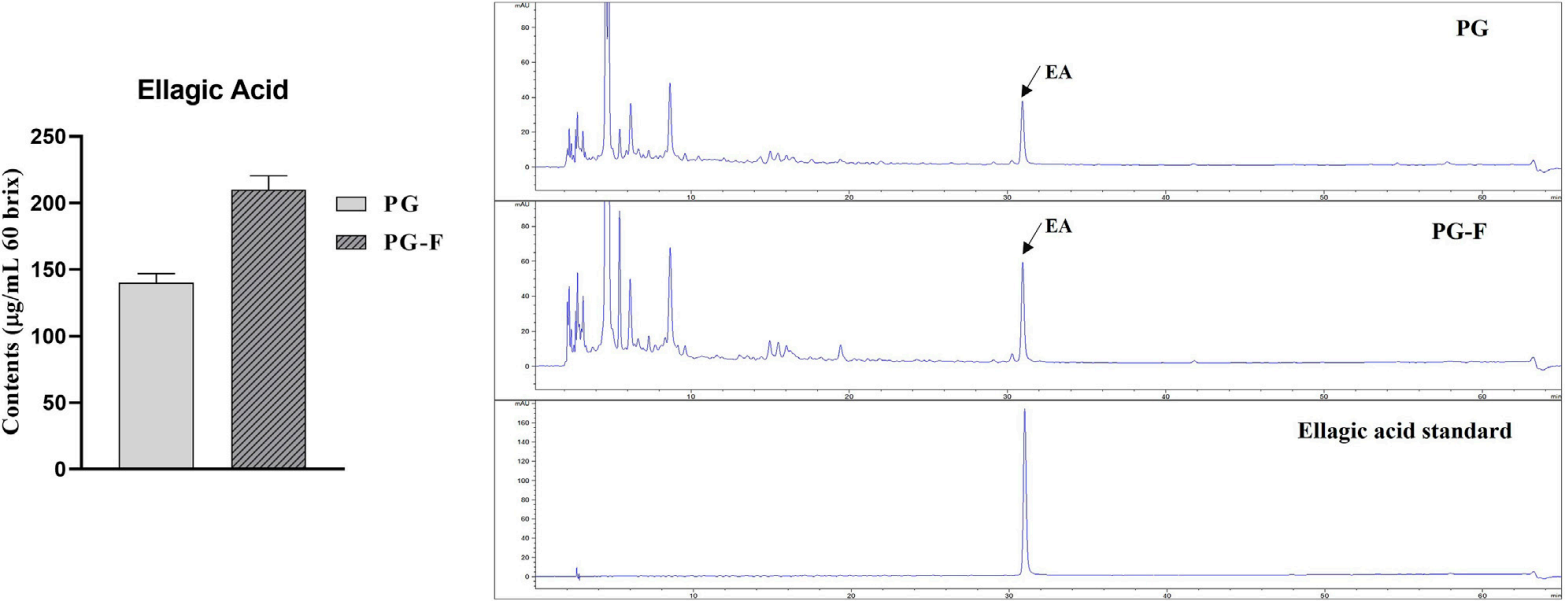
Total amount of ellagic acid in Pomegranate (fermented and unfermented).

Ellagic acid (EA), a phenolic phytochemical, is one of the most critical components of fruits and vegetables (Vattem and Shetty, 2005). Undoubtedly, ellagic acid is a crucial antioxidant and responsible for other pharmacological effects, including cancer, cardiovascular diseases, and inflammation (Ríos et al., 2018). Based on many reports, ellagic acid is Pomegranate’s most important bioactive compound, which works against mutagen and carcinogens, heart diseases, atherosclerosis, wound healing, and skin elasticity (Seeram et al., 2005; Moccia et al., 2019). Mostly EA is present in pomegranates in a meager amount. It is mainly present as a complex form of ellagitannins, punicalagin isomers, and granatin, which can be liberated into monomolecular substances with high physiological activity; there are physicochemical and biological methods, but biotechnology using enzymes is effective (Mena et al., 2014; Garcia-Villalba et al., 2015). However, according to our results, fermentation process increased the amount of ellagic acid in Pomegranate. When PG juice was treated with TAH enzyme 0.1% for 2 days along with *Lactobacillus vespulae* DCY75, EA increased by 70% (Figure 2). Different studies have been carried out to increase the health benefits of pomegranate juice by the fermentation process. For example, the best probiotic lactic acid bacteria were selected by evaluating the growth rate during the fermentation of pomegranate juice as a carbon source (growth factors) and the viability under low-temperature storage conditions (Mousavi, Mousavi et al., 2011) whereas our study focused on selecting a strain that promotes the production of ellagic acid, a useful ingredient in pomegranate juice. Furthermore, HPLC analysis was carried out to determine the levels of EA in the juice from the fermented and unfermented pomegranate juice. The results have shown that fermented Pomegranate has increased the ellagic acid profile compared to unfermented Pomegranate (Figure 2).

### 3.3 Total phenolic and total flavonoid contents

Phenolics and flavonoids are the largest phytochemicals group that possess antioxidant activity in fruits, vegetables, and plants (Ribarova, Atanassova et al., 2005). They also exhibit effects against ulcer, inflammation, tumor, depression, and cancer (Huyut, Beydemir et al., 2017). To estimate the total phenolic content (TPC) and total flavonoid content (TFC) of the pomegranate juice, the Folin–Ciocalteu and aluminum chloride colorimetric methods were carried out, respectively.

The phenolic contents vary, ranging from 0.31 ± 0.01 to 0.45 ± 0.01 mg/g, expressed as gallic acid equivalents (GAE). The flavonoid contents vary from 0.11 ± 0.04 to 0.18 ± 0.02 mg/g, expressed as rutin equivalents (RE). A previous report also reported that the TPC has increased by fermentation (Ríos et al., 2018). In this study, phenolic and flavonoid contents eventually increased in fermented PG compared to unfermented PG. The amount of TPC has significantly increased by 0.14 ± 0.01 mg/g FW in PG-F than PG (Table 3).

**TABLE 3:** Total phenolic and total flavonoid contents.

Pomegranate	TPC	TFC
PG-Control	mg GAE/g FW	mg RE/g FW
	0.31 ± 0.01	0.11 ± 0.04
PG-F	0.45 ± 0.01	0.18 ± 0.02

### 3.4 Antioxidant activity: DPPH and reducing power assays

Synthetic antioxidants with low cost and bland flavor have been used for decades as chemicals for food storage and the prevention of the oxidation process. Various assays are available to measure antioxidant activity. Among those, we selected DPPH and the potassium ferricyanide reducing power assay to quantify the antioxidant activity of our samples expressed as gallic acid equivalents (GAE) and ascorbic acid equivalents (AAE). Moreover, these assays are generally used to determine the antioxidant potential of different compounds as free radical scavengers or hydrogen donors (Warinhomhoun, Muangnoi et al., 2021).

The DPPH results expressed that the antioxidant capacity of unfermented PG ranged from 1.29 ± 0.02 mg GAE/g FW to 3.71 ± 0.04 mg RE/g FW and 3.71 ± 0.03 mg AAE/g FW. The DPPH scavenging activity of fermented PG ranged from 1.45 ± 0.03 mg GAE/g FW, 3.92 ± 0.03 mg RE/g FW, and 4.15 ± 0.04 AAE/g FW, which shows PG-F has better antioxidant activity than PG. Similarly, the result of the reducing power assay revealed that the antioxidant power of PG ranged from 4.12 ± 0.12 mg GAE/g FW, 12.99 ± 0.12 mg RE/g FW to 11.13 ± 0.05 mg AAE/g FW, and the PG-F ranged from 4.64 ± 0.13 mg GAE/g FW, 13.82 ± 0.02 mg RE/g FW to 12.45 ± 0.08 mg AAE/g FW. The result clearly shows the increase in antioxidant capacity of fermented Pomegranate (Table 4).

**TABLE 4:** Potential antioxidant activities of Pomegranate.

Pomegranate	*In Vitro* antioxidant						
		DPPH			Reducing power		
PG	mg GAE/g FW	mg RE/g FW	mg AAE/g FW	mg GAE/g FW	mg RE/g FW	mg AAE/g FW
	1.29 ± 0.02	3.71 ± 0.04	3.71 ± 0.03	4.12 ± 0.12	12.99 ± 0.12	11.13 ± 0.05
PG-F	1.45 ± 0.03	3.92 ± 0.03	4.15 ± 0.04	4.64 ± 0.13	13.82 ± 0.02	12.45 0.08

### 3.5 PG-F increased the proliferation of human MCF-7 cells

The MCF-7 cell proliferation assay assesses the cellular response in estrogenic or antiestrogenic compounds in an ER-mediated pathway (Ahn, Jeong et al., 2014). Pomegranate (PG) juice was investigated for its ability to increase the cell viability of estrogen-dependent MCF-7 cells. The MCF-7 cells were commonly used in detecting estrogen-like activity. Moreover, cells treated with estrogen-like substances can promote the proliferation of estrogen receptor-positive cells MCF-7 (Ribarova 2005).

As we know, MTT is a ubiquitously used tool to measure toxicity *in vitro* but it has some merits and demerits. According to (Ghasemi, Turnbull et al., 2021) MTT assay measurement is affected by cell number, MTT concentration, and MTT incubation time. It is essential to optimize these parameters for each cell line. Additional optimization of experiments is cost-effective, tiresome, and time killing, yet fundamental. In spite of the limitations, many previous studies have used the MTT assay to examine cell proliferation in MCF7 cells and cell viability of different cells (Hu, Hou et al., 2007; Wang et al., 2018; Tanaka, Onuma et al., 2019; Wang et al., 2021).

**FIGURE 3:**
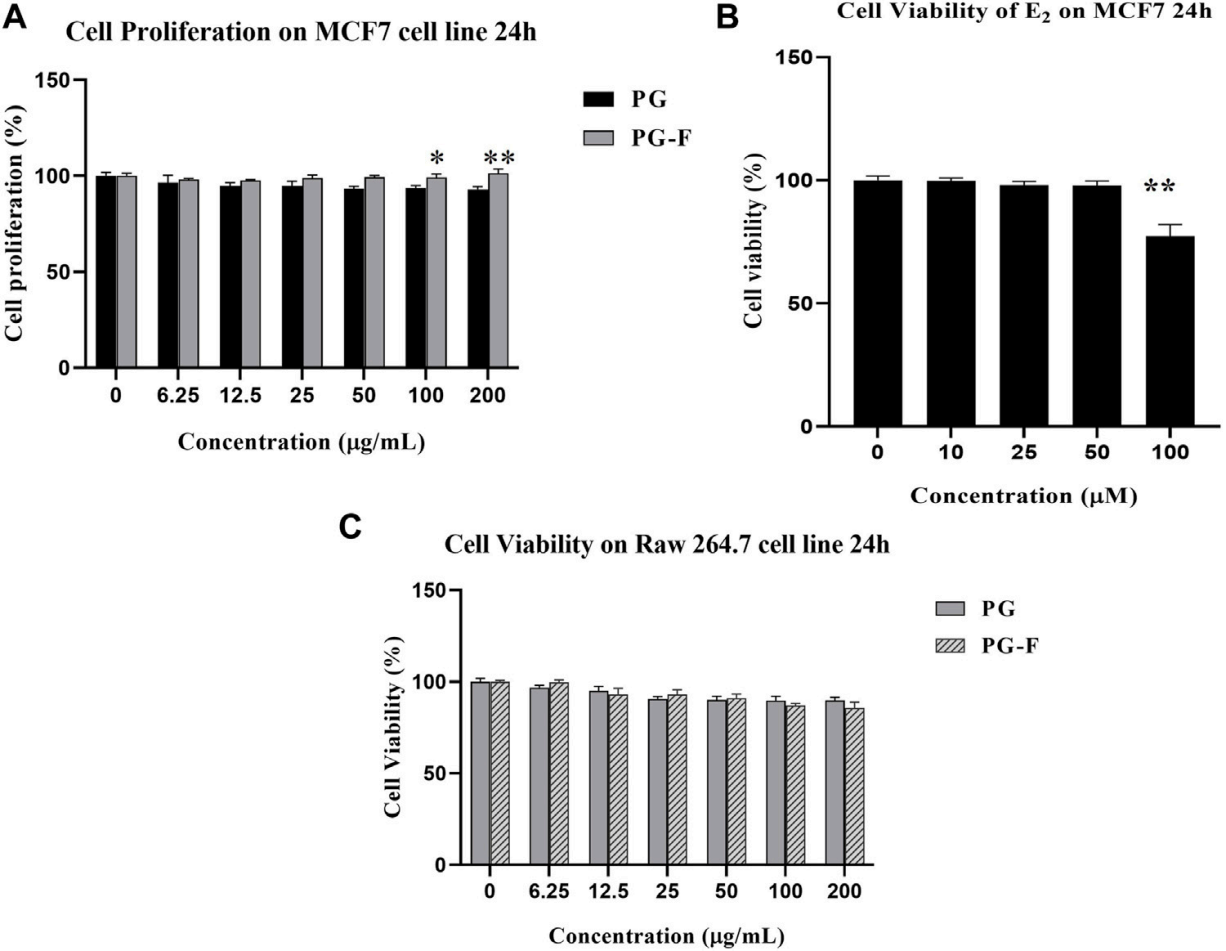
Cell proliferation assessment **(A)** using MTT in MCF7 cells. The data shown represent the mean values of three experiments ±SD. *p < 0.05, **p < 0.01 as compared with the PG treated group **(B)** Cell viability of various concentrations of estradiol on MCF7 cells. **p < 0.01, as compared with the non-treated group. **(C)** Cell viability measurement of RAW 264.7 cells following the incubation of various concentrations of Pomegranate for 24 h.

In the current study, we have used MTT assay to determine the cell proliferation in MCF7 cells. And the result showed that Pomegranate and fermented Pomegranate juice (6.25–100 μg/ml) have also increased cell proliferation (Figure 3A). Compared to Pomegranate, fermented Pomegranate has increased cell viability more significantly. Estradiol (E_2_) was used as an ER agonist (positive control) in estrogen-derived cell proliferation in MCF-7 cells. E_2_ has not shown any cytotoxicity until 50 µM (Figure 3B) but exerted toxicity at 100 µM. So, we selected E_2_ (50 µM) for further experiment, whereas 100 μg/ml of juice was chosen for both the pomegranates (PG and PG-F). Besides, both PG and PG-F juice has not shown any significant change in the cell viability of Raw 264.7 cells (Figure 3C).

### 3.6 Effect of Pomegranate on the lipopolysaccharide induced Nitric oxide production

Macrophages play an essential role in inflammatory diseases associated with the excessive production of inflammatory mediators, such as NO, PGE2, iNOS, and COX-2 (Kim et al., 2018). The most dominant inflammatory mediator is Nitric oxide (NO), a signaling molecule which plays a key role in the pathogenesis of inflammation (Rath et al., 2014). In normal conditions, it gives an anti-inflammatory effect, but the overproduction of NO is considered as a pro-inflammatory mediator of inflammation (Bian and Murad, 2003). The overproduction of nitric oxide happens in abnormal situations such as inflammatory bowel diseases, arthritis, osteoporosis, and other inflammatory diseases of the respiratory system (Akanji et al., 2020). Consequently, subjugating NO overproduction has become an influential target in treating inflammatory disorders. Since estrogens have an anti-inflammatory role, the risks of inflammation increase with decreased estrogen levels in the postmenopausal state (Aviv, Valdes et al., 2006; Christensen and Pike 2015; Park, Lee et al., 2016).

**FIGURE 4:**
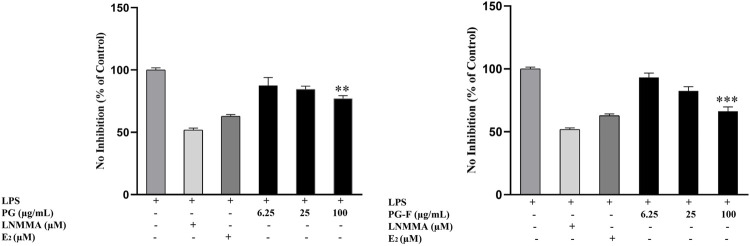
Effects of Pomegranate on the NO inhibition. RAW 264.7 cells were pretreated with Pomegranate juice for 1 h and then stimulated with LPS (1 μg/ml) for 24 h. The concentrations of nitrite were measured as described in the materials and methods. The data shown represent the mean values of three experiments ±SD. **p < 0.01, ***p < 0.001 as compared with the group treated with LPS.

We examined the anti-inflammatory effect of Pomegranate on Raw 264.7 cells. Cells were treated with both Pomegranates types (PG and PG-F), followed by lipopolysaccharide (LPS) (1 μg/ml) for 24 h. Our study used a common nitric oxide inhibitor, L-NMMA, as a positive control. As shown in Figure 3, NO production is significantly higher in LPS-treated cells, whereas in Pomegranate treated LPS-induced cells, NO production has decreased in a dose-dependent manner (Figure 4).

As we know, PG-F contains a high amount of ellagic acid that has already been proven to have a significant anti-inflammatory effect (Mousavi et al., 2011). Moreover, fermented pomegranate juice has an ample amount of total phenolic and flavonoids. Previous studies have also shown that phenolics/flavonoids can exert an anti-inflammatory effect *via* inhibiting NO production as well as suppressing intracellular cytokines (Zhang, Ravipati et al., 2011; Hong, Pangloli et al., 2020). Fermented Pomegranate has shown better anti-inflammatory effects in comparison with unfermented Pomegranate juice.

### 3.7 Pomegranate increased the estrogen receptors mRNA expression and estrogenic activity in human MCF-7 cells and suppressed inducible isoform in lipopolysaccharide-induced RAW 264.7 macrophages

The subtypes of estrogen receptors (ERα and ERβ) highly modulate the physiological functions of estrogenic compounds. ERα is found mainly in the ovary, mammary gland, uterus, male reproductive organs, and adipose tissue. In contrast, ERβ is present in the colon, the prostate, bladder, ovary, adipose tissue, and immune system (Paterni, Granchi et al., 2014). The transcriptional effects of estrogen are mediated by two key estrogen receptors (ER), ER alpha (ERα) and ER beta (ERβ). These cells regulate the uterus morphological changes in response to the circulating estrogen concentrations. To determine the estrogen-like activity of Pomegranate, we measured the expression level of several genes that play an essential role in regulating the reproductive system. ERα, ERβ, and the estrogen-regulated gene pS2 present in breast cancer cell line MCF7 were chosen primarily for this study. A considerable number of studies have chosen ER subtype mediated pathway to study phytoestrogenic activity of desired compounds both *in vitro* and *in vivo* (Klinge, Risinger et al., 2003; Oh and Chung 2004; Mishima, Suzuki et al., 2005; Nanashima, Horie et al., 2015; Xu, Ding et al., 2016). For good measure, the inducible isoform (iNOS) is a major downstream mediator of inflammation in various cell types including immune cells, fibroblasts, endothelial cells, and skeletal muscle cells. Besides, iNOS produces large amounts of NO as a defense mechanism (Nakazawa, Chang et al., 2017). And overproduction of NO by iNOS can inhibit energy production, cause direct injury to the mitochondrial respiratory machinery as well as causes inflammation.

**FIGURE 5:**
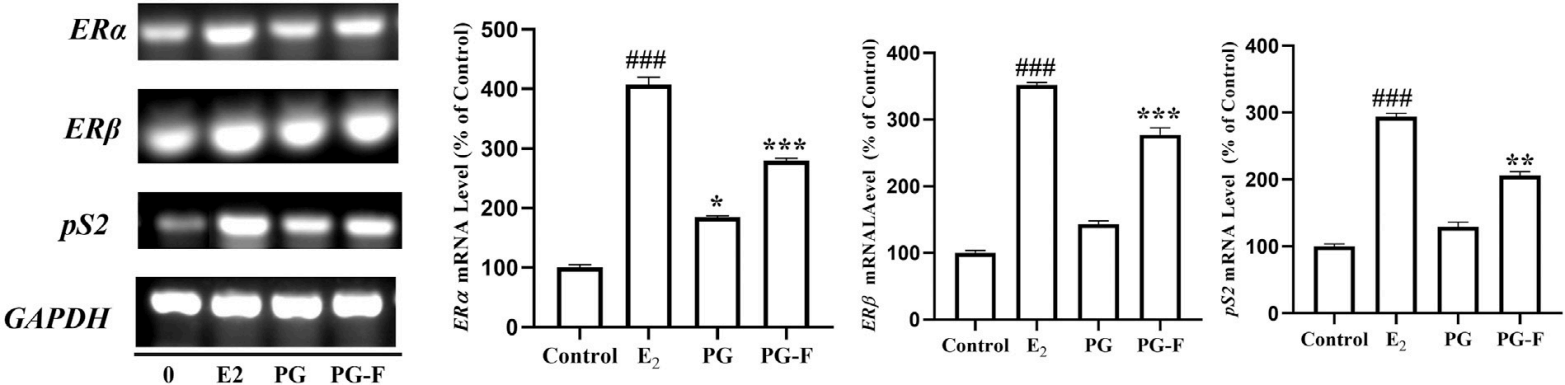
Effect of Pomegranate on the transcriptional activation of the ER α, Erβ, and pS2 genes in MCF7 cells. MCF7 cells were treated with Pomegranate juice for 24 h. Subsequently, total RNAs were extracted, and the mRNA expression levels were determined by RT-PCR analysis and compared with those of GAPDH. The data shown are representative of the mean values of three independent experiments ±SD. *p < 0.05, **p < 0.01, ***p < 0.001 as compared with the group treated with E2, and ###*p* < 0.001 as compared to the control.

The gene expressions were analyzed using RT-PCR to evaluate the estrogen-like activity of the samples. Our results revealed that both PG and PG-F increased the expression of ERα and ERβ in MCF7 cells. Both of the genes expressed significantly when compared with the estradiol expression. The ERβ expression was higher than that of ERα expression. In addition, both pomegranate and fermented pomegranate upregulated the expression of the estrogen-sensitive gene pS2. However, all of the genes (ERα, ERβ, pS2) were more highly expressed by the treatment of PG-F than PG treatment (Figure 5). In addition, many researches have demonstrated that flavonoids/phenolics possessed biological activity as estrogens (Miksicek, 1995; Yang, Allred et al., 2012; Tungmunnithum, Thongboonyou et al., 2018). As mentioned, PG-F has increased amount of total phenolic/flavonoids content along with high index of ellagic acid, it has shown more preferable estrogenic effect than PG via ERα, ERβ mediated pathway.

**FIGURE 6:**
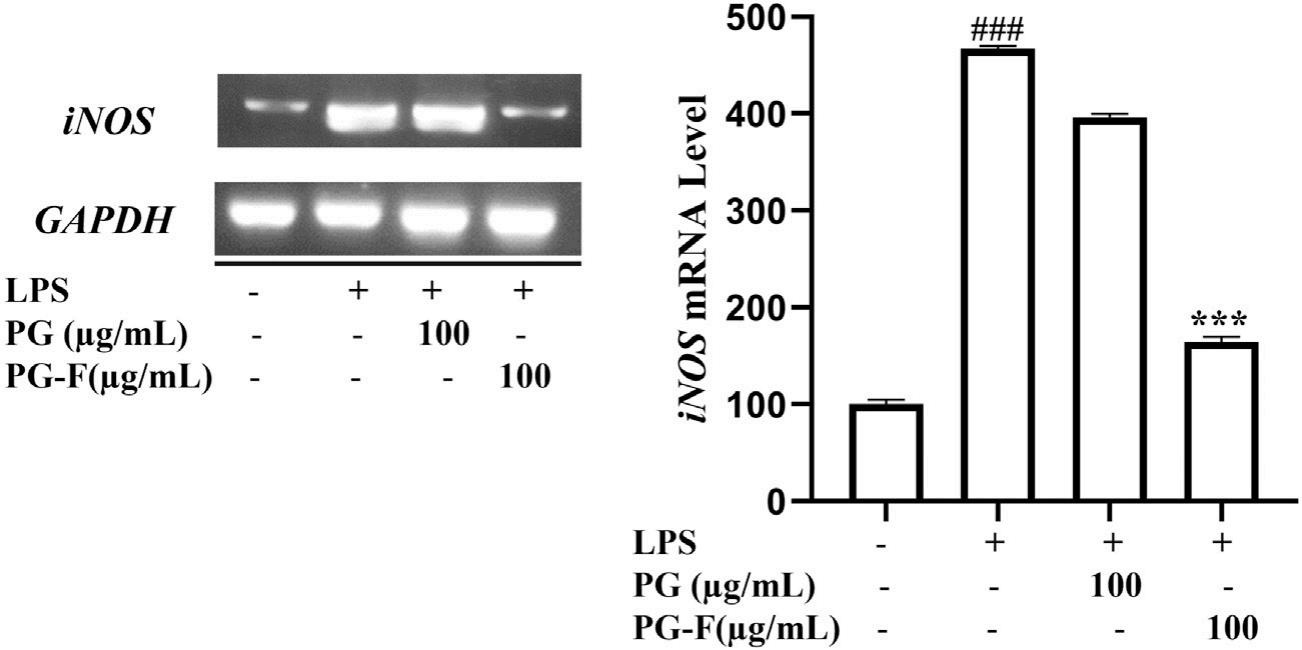
Effect of Pomegranate on the mRNA expression of iNOS in RAW 264.7 cells. AW 264.7 macrophages were pretreated with Pomegranate juice for 1 h, then stimulated with LPS (1 μg/ml) for 24 h. Finally, total RNA was extracted, and the mRNA expression levels were determined by RT-PCR analysis and compared with those of GAPDH. The data shown are representative of the mean values of three independent experiments ±SD. ***p < 0.001 as compared with the group treated with LPS, and ###*p* < 0.001 as compared to the control.

Our result supports the estrogen-like activity of PG-F by promoting MCF7 cell proliferation and upregulation of ER subtypes. On the other hand, iNOS expression was suppressed by the PG-F treated LPS stimulated Raw 264.7 cells (Figure 6). This supports the lower NO production leading to the anti-inflammatory effect of our samples.

## 4 Conclusion

In the present study, an increased amount of EA was obtained from the fermented pomegranate juice, where the fermentation was carried out through TAH and microbe *Lactobacillus vespulae* DCY75 for a high yield of EA. HPLC analysis has shown the difference between the EA yield in fermented and unfermented PG. The EA was significantly higher in fermented Pomegranate. On top of that, fermentation has escalated the level of total flavonoids and phenolics present in pomegranate juice. Correspondingly, the antioxidant activity of PG and PG-F was measured, and it was found that PG-F has higher antioxidant properties. While comparing both the samples, PG-F increased ER receptor expression more significantly than PG. In addition, a low level of estrogen is a crucial reason for inflammation. We measure the NO inhibition and iNOS gene expression in RAW 264.7 cell line. The fermented Pomegranate has reduced NO production dose-dependently and suppressed iNOS significantly, which possesses anti-inflammatory activity of PG-F.

## Data Availability

The raw data supporting the conclusions of this article will be made available by the authors, without undue reservation.
